# Evidence of emotion-antecedent appraisal checks in electroencephalography and facial electromyography

**DOI:** 10.1371/journal.pone.0189367

**Published:** 2018-01-02

**Authors:** Eduardo Coutinho, Kornelia Gentsch, Jacobien van Peer, Klaus R. Scherer, Björn W. Schuller

**Affiliations:** 1 Department of Music, University of Liverpool, Liverpool, United Kingdom; 2 Department of Computing, Imperial College London, London, United Kingdom; 3 Swiss Center for Affective Sciences, University of Geneva, Geneva, Switzerland; 4 Behavioural Science Institute, Radboud University Nijmegen, Nijmegen, Netherlands; 5 Chair of Complex & Intelligent Systems, University of Passau, Passau, Germany; Universita degli Studi di Pisa, ITALY

## Abstract

In the present study, we applied Machine Learning (ML) methods to identify psychobiological markers of cognitive processes involved in the process of emotion elicitation as postulated by the Component Process Model (CPM). In particular, we focused on the automatic detection of five appraisal checks—novelty, intrinsic pleasantness, goal conduciveness, control, and power—in electroencephalography (EEG) and facial electromyography (EMG) signals. We also evaluated the effects on classification accuracy of averaging the raw physiological signals over different numbers of trials, and whether the use of minimal sets of EEG channels localized over specific scalp regions of interest are sufficient to discriminate between appraisal checks. We demonstrated the effectiveness of our approach on two data sets obtained from previous studies. Our results show that novelty and power appraisal checks can be consistently detected in EEG signals above chance level (binary tasks). For novelty, the best classification performance in terms of accuracy was achieved using features extracted from the whole scalp, and by averaging across 20 individual trials in the same experimental condition (*UAR* = 83.5 ± 4.2; *N* = 25). For power, the best performance was obtained by using the signals from four pre-selected EEG channels averaged across all trials available for each participant (*UAR* = 70.6 ± 5.3; *N* = 24). Together, our results indicate that accurate classification can be achieved with a relatively small number of trials and channels, but that averaging across a larger number of individual trials is beneficial for the classification for both appraisal checks. We were not able to detect any evidence of the appraisal checks under study in the EMG data. The proposed methodology is a promising tool for the study of the psychophysiological mechanisms underlying emotional episodes, and their application to the development of computerized tools (e.g., Brain-Computer Interface) for the study of cognitive processes involved in emotions.

## Introduction

Research in the affective sciences aims at understanding the mechanisms driving human emotion (and related processes). Although a definition of emotion that all emotion researchers would agree on is lacking (see e.g., [1–3]), emotions can generally be described as responses to events that are important to an individual, and typically include cognitions, action tendencies, bodily responses, expression and subjective feelings (see e.g., [1, 3, 4]). A prominent set of theories in this area that attempts to explain the cause and variation of emotions are the so called “appraisal” theories. Appraisal theorists consider event evaluation (appraisal) to be the core mechanism of emotion elicitation and differentiation. They conceptualize emotion as an emergent, dynamic process, initiated by the individual’s subjective appraisal of events (see [5–8]).

Appraisal refers to cognitive mechanisms that rapidly judge the personal impact of emotion-evoking objects, events, or situations. Several appraisal criteria (e.g., novelty, pleasantness, goal conduciveness, and coping potential) operate to assess the impact of an event on the individual (e.g., [8]). The unique combination of the outcomes for the different appraisal criteria determines the type and intensity of the elicited emotion(s). This outcome will in turn orchestrate a series of (coordinated) responses in the so-called emotion components such as motivation (e.g., approach or avoidance), bodily responses (e.g., cardiovascular changes), expression (facial, vocal, and gesture), and subjective feelings (the conscious experience of an emotion) (see [5] for an overview). The role of appraisal processes in the elicitation and differentiation of emotional episodes has been incorporated in the main theoretical approaches to emotion (including modern work in the basic emotion and constructivist traditions, see e.g., [9, 10]; also, see [3] for a review). However, in the current work, we focus on appraisal models, as these make the most specific predictions about these cognitive mechanisms.

### The Component Process Model (CPM)

**Fig 1:**
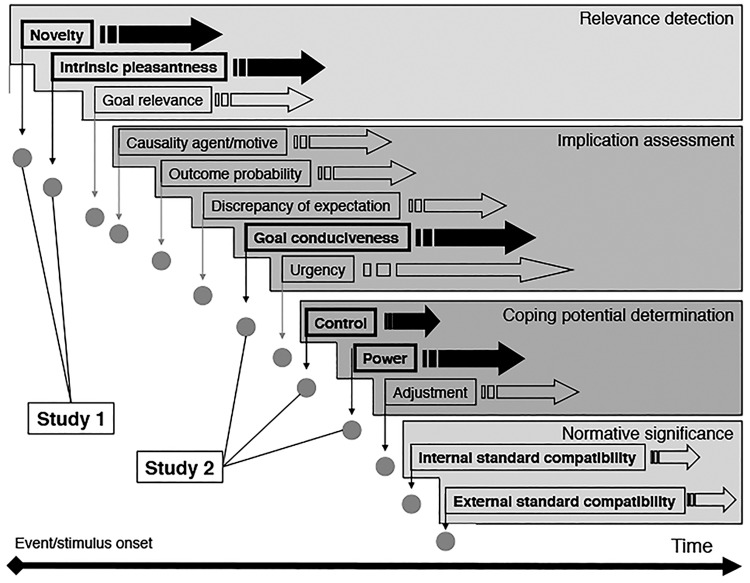
Component Process Model. The Component Process Model (CPM, e.g., [5]) describes a functional architecture of the appraisal process.Several appraisal checks (each evaluating specific information of an event) assess in a fixed sequence the overall significance of an event at four major levels: *relevance* of the event for the individual; *implications* or consequences of the event; *coping potential* how well the individual can cope with or adjust to these implications; and *normative significance* of the event.

A prominent appraisal model of emotion that proposes a framework representing and operationalizing the components and functions of emotion as a psychobiological and cultural adaptation mechanism is the CPM ([5]). The CPM describes a functional architecture of the appraisal process. Specifically, several major so-called *stimulus* appraisal checks (SECs), each evaluating specific information concerning the emotion-eliciting event, assess the overall significance of an event in a fixed sequence (see Fig 1). First, the relevance of the event for the individual is assessed. Second, the implications or consequences of the event, and how they affect the individuals well-being are inferred. Next, coping potential estimates how well the individual can cope with or adjust to these implications. Lastly, the normative significance of the event for the individual is appraised (i.e., the impact of the event on self-concept, internalized social norms and values). At each moment individuals rapidly evaluate events on the bases of these major appraisal checks. Importantly, each major appraisal check determines a number of specific subordinate appraisal criteria. For example, subordinate appraisal criteria for relevance detection are *novelty*, *intrinsic pleasantness* (or *valence*) and *goal relevance*; for implication assessment *causal attribution*, *outcome probability*, *discrepancy-from-expectation*, *goal conduciveness*, and *urgency*; for coping potential determination *control*, *power*, and *adjustment*. These appraisal checks are sequentially and recursively processed, and their results are cumulatively integrated into a specific emotional response pattern which is centrally represented and often reaches consciousness. In other words, the profile of an appraisal check sequence offers unique information about the quality and the intensity of the emotional state of an individual person. Furthermore, the CPM holds that the outcome of each (subordinate) appraisal check triggers a cascade of efferent effects to the autonomic nervous system (e.g., cardiovascular and respiratory changes) and the somatic nervous system (causing muscular activity changes which become evident as emotional expressions in face, voice, and body).

### Analysis of brain and facial muscle activity

Although emotion theories offer very detailed explanations about appraisal processes, these are typically phenomenological and very little is known about the actual biological substrates supporting them. Research aimed at better understanding the cognitive and psychobiological processes underlying emotion elicitation and expression often involves the study of patterns in brain activity (electroencephalography; EEG) and facial muscle activity (facial electromyography; facial EMG) (see [11] for a review of methods commonly used for the measurement of emotion). Such data allows advancing the systematic testing of emotion theories and the development of sophisticated tools for the assessment of (deviations in) emotional processing. Recently, such methods have also been used to provide empirical evidence for the CPM (see [12] for an overview).

However, the analysis of EEG and facial EMG data poses a number of important methodological challenges: In addition to issues related to high dimensionality (i.e., large number of signals), both types of signals are characterized by non-stationarity, low signal-to-noise ratios (SNRs), and large trial-to-trial and participant-to-participant variability. As a means to deal with these challenges of physiological data analysis, traditional neuroscientific analysis strategies are based upon averaging methods (i.e., calculating the grand average over trials of an experimental condition) to eliminate random noise and to enhance signal distinctiveness (i.e., SNRs). This approach is costly and time-consuming as it often requires hours of recordings (e.g., a minimum number of trial repetition is necessary to reduce noise sufficiently through averaging). This may affect the processes under investigation, for example, due to habituation, fatigue, or learning. Moreover, extensive EEG and facial EMG recordings are not always possible, especially in the case of specific populations (e.g., babies, children, or patients with mental or neurological conditions). The challenge is thus to create new analysis strategies that allow a robust data-driven identification and discrimination of relevant information in EEG and EMG signals based on a restricted number of trials.

In this context, Machine Learning (ML) methods are a promising technique. ML is a sub-field of Computer Sciences focused on the study and creation of algorithms that can learn from and make predictions on data [13], and permitting computers to learn without being explicitly programmed [14]. Using ML techniques computers learn by searching for distinct patterns in data. This helps them to deal with the challenges of central and peripheral physiological signals without requiring *a priori* decisions about the analyses of the EEG and EMG recordings.

### Overview of this paper

In this paper, we describe an application of ML to the detection of EEG and facial EMG signal patterns related to the processing of appraisal checks. Whereas ML techniques have been applied to the recognition of user states (including affective states) from EEG [15] and EMG [16] signals, this is the first time that ML is being used to identify evidence of fine-grained information of emotional processes.

Using two data sets from previous studies [17, 18] designed to examine predictions of the CPM about the processing and efferent effects of appraisal checks, our aims are three-fold: First, we want to determine whether experimental manipulations of appraisal checks (e.g., the detection of a novel event vs. a familiar one) are consistently detectable in EEG and EMG activity using ML techniques. In particular, having in mind the application of this work to the automatic detection of appraisal checks in new data, we focus on a participant-independent scenario, that is, creating models by detecting patterns from a subgroup of all participants, and testing them in signals from a new subgroup of participants.

**Fig 2:**
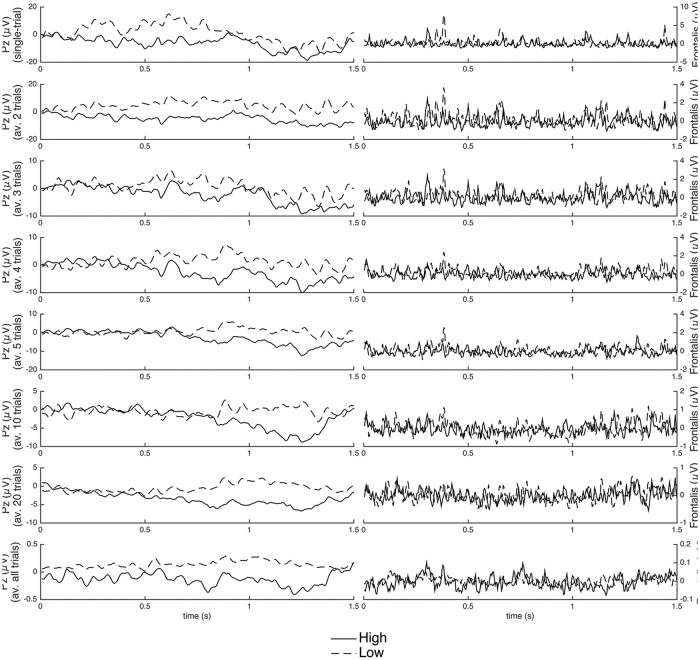
Typical EEG and EMG signals. Typical EEG (*Pz* electrode signal; left) and facial EMG (*Frontalis* muscle signal; right) signals for the two contrasting conditions (“high” vs. “low”) of the *Control* appraisal check (one participant from Study 2). The signals are shown for single trials and the average signal over 2, 3, 4, 5, 10, 20 and all trials (example data from [17]).

Second, we aim to identify the minimal number of trials of an experimental condition necessary for a successful classification by the created ML models, using the single-trial recordings and the average recordings over different numbers of trials (2, 3, 4, 5, 10, 20, and all trials; see Fig 2). Third, we explore whether a small set of EEG channels over specific scalp regions (e.g., midline frontal and parietal) that have been previously shown to be associated with appraisal processing is sufficient to detect signals related to specific appraisal checks (and therefore, could reduce the complexity of an experimental setup).

## Data sets

The data in this work were taken from two previous studies ([17, 18]) that addressed three fundamental questions regarding the mechanisms underlying the appraisal process: Whether appraisal criteria are processed (a) in a fixed sequence, (b) independently of each other, and (c) by different neural structures or circuits. In Study 1 ([17]), an oddball paradigm with affective pictures was used to experimentally manipulate novelty and intrinsic pleasantness appraisal checks. This data set includes EEG and facial EMG recordings from twenty-six subjects. In Study 2 ([18]), a gambling task was applied in which feedback stimuli manipulated simultaneously the information about goal conduciveness, control, and power appraisals. This data set includes EEG and facial EMG recordings from twenty-four subjects. In both studies, EEG was recorded during task performance, together with facial EMG, to measure, respectively, cognitive processing and efferent responses stemming from the appraisal manipulations. Full details about the studies can be found in the original publications. The complete data sets as well as a full description (including pre-processing procedures) are freely available [19, 20]In the following subsections, we provide the core information of the two data sets with relevance to this article.

### EEG recordings and pre-processing

The EEG was recorded at 512 Hz with a Biosemi Active-Two system (BioSemi Biomedical Instrumentation, Amsterdam, the Netherlands) from 64 active electrodes referenced to an active common mode sense (CMS) and with a passive driven right leg (DRL) ground electrode. All electrodes were mounted in an elastic cap and evenly distributed over the head surface according to the international extended 10–20 system.

#### Study 1

In this study [17] an oddball paradigm with affective pictures was used to experimentally manipulate novelty and intrinsic pleasantness appraisal checks. Signals were pre-processed offline using Brain Vision Analyzer software (version 2.0, Brain Products, Gilching, Germany). Bad channels were interpolated using a topographic interpolation (using spherical spline; [21]), with a maximum of six channels for each individual data set. Interpolation affected channels from the smallest set (EEG channels of interest; see Section Computational experiments for more details) in only one participant, and channels from the second set (those of interest plus the surrounding 13 channels) in only four participants. Overall, only 2.1% of the total data (25 participants x 64 channels) were interpolated. Finally, data were downsampled to 256 Hz with a spline interpolation, filtered (high pass: 0.1 Hz, 24 dB/oct; low pass: 30 Hz, 48 dB/oct), and re-referenced to an average reference including all electrodes.

Next, data were segmented into epochs ranging from -200 to +800 ms relative to stimulus onset, based on codes synchronized to stimulus presentation. All segments were corrected for the effects of eye blinks and eye movements using a standard procedure [22], and segments including motor responses or artifacts (amplitude values larger than 75 *μV*, a difference > 100 *μV* between the lowest and the highest amplitude within the segment, a period > 100 *ms* with activity < 0.50 *μV*, or a difference > 50 *μV* between two subsequent sampling points) were excluded.

Finally, baseline (-100 to 0 ms relative to stimulus onset) corrected data of the post stimulus time interval were exported for all remaining segments of the six relevant experimental conditions (2 novelty × 3 intrinsic pleasantness). The final number of EEG trials retained and used in our study (across all participants and conditions) amounts to 16666. Channels of interest were the three midline electrodes (Fz, Cz, Pz), for the P3 and the late positive potential (LPP) event-related potential (ERP) components.

#### Study 2

In this study [18], a gambling task was applied in which feedback stimuli manipulated simultaneously the information about goal conduciveness, control, and power appraisals. Signals were pre-processed offline. First, they were downsampled to 256 Hz using the Biosemi decimeter software package (BioSemi Biomedical Instrumentation, Amsterdam, Netherlands). Next, in EEGLAB (version 11.0.4.3b; [23]), implemented in Matlab R2012a (The MathWorks, Inc., Natick, MA), the data were high-pass filtered (0.1 Hz), noisy channels were removed, horizontal and vertical eye movements were corrected (based on individual component maps, extracted by Infomax independent component analysis implemented in EEGLAB (see [24]). Then, the data were exported to Brain Vision Analyzer software (BVA, Brain Products, Gilching, Germany). In BVA, the spherical spline interpolation of channels, low-pass filtering 30 Hz and segmentation (-200 ms pre-stimulus and 1500 ms post-stimulus) were performed similar to Study 1. Interpolation affected a channel from the smallest set (four EEG channels of interest) in only three participants, and one or two channels from the second set (16 channels) in nine participants. Overall, only 4.4% of the total data (24 participants x 64 channels) was interpolated. Finally, trials in which artifacts exceeded ±110 *μV* were removed (2.62% total amount of excluded trials across all participants).

Finally, the segmented data were baseline corrected (-200 to 0 ms relative to stimulus onset) and single trials were separated according to their experimental condition. The final number of EEG trials retained in Study 2 and used in the present analyses (across all participants and conditions) amounts to 20185. Channels of interest were Fz and FCz for the feedback-related negativity ERP component, and Pz and POz for the P3 ERP component.

### Facial EMG recordings and pre-processing

The similar data acquisition and pre-processing steps were applied in both studies. Facial EMG was recorded from six electrodes (Study 1: using a Biopac amplifier system and Study 2 using the Biosemi EMG electrodes). All electrodes were attached to the left side of the face, corresponding to three distinct bipolar montages over the medial frontalis, the corrugator supercilii, and the zygomaticus major muscle regions [25].

Signals were pre-processed offline using MATLAB software (version 7.12.0.635, The MathWorks, Inc., Natick, MA, USA). All data were band pass filtered from 20-400 Hz, rectified, smoothed with a 40 Hz low pass filter, and downsampled to 256 Hz. Next, data were segmented into epochs ranging from 0 to 1,500 ms relative to stimulus onset, based on codes synchronized to stimulus presentation.

Then, the distribution of EMG values for each muscle region was closely inspected for outlying values. Given the lack of established methods in the literature, EMG trials were evaluated based on the range of values (maximum—minimum) for each muscle region. Outlying trials were identified using a threshold of twice the upper 75th percentile value of ranges (over all individual trials across participants and conditions) for each muscle region. This level seemed to provide a good balance between excluding clearly divergent recordings (e.g., trials contaminated by movement artifacts) while still including relatively large reactions that contain an important signal of the manipulated appraisal checks. Any trial whose range was greater than this value, for any of the three muscle regions, in either the baseline or post-stimulus period, was removed. If any participant had over 50% of total trials outlying, all trials for that participant were removed (this was the case for two participants). This decision was motivated by considering that this excessive activity could be due to movement artifacts and/or misplacement of the EMG electrodes.

Finally, all trials were baseline corrected in relation to the average of the pre-stimulus period of 100 ms, and only the post-stimulus period of 1500 ms was exported for further analysis. The final number of EMG trials retained amounts to 21529 (Study 1) and 18480 (Study 2).

## Computational experiments

In this section, we describe the computational experiments conducted to model each appraisal check independently. Given that the EEG and EMG recordings for each experimental condition consisted of the simultaneous manipulation of two (Study 1: novelty and intrinsic pleasantness) and three (Study 2: goal conduciveness, control, and power) appraisal checks, the original experimental conditions were converted into appraisal check-specific three (intrinsic pleasantness: three levels) and two (all other appraisal checks: two levels) class problems. In this way, all available trials were used for the development of a ML classifier for each appraisal check. In this context, a ML classifier is an algorithm developed to identify to which set of categories (or sub-populations) a given observation (hereafter named instance) belongs, by using a training set of data containing observations (i.e., instances) whose category membership is known. Using Study 1 as an example, all trials involving the presentation of “novel” stimuli (outcome of the novelty check) were labeled as “novel” irrespective of the manipulated intrinsic pleasantness check (negative, neutral, or positive).

In order to investigate the impact of averaging different numbers of trials on the classification performance, and to identify the minimal number of trials necessary to achieve the best possible classification results, we created different classifiers to discriminate the outcome of the various appraisal checks from single trials and averaged trials (2, 3, 4, 5, 10, 20, and all trials available for each class). For each number of averaged trials *T*, the average of each EEG and EMG channel signal of each participant was computed for all groups of *T* trials available for a specific class. When the number of available trials for a specific class is not a multiple of *T*, the remaining trials were discarded. The number of instances available for each classification experiment is shown in Table 1. In Table 2, we indicate the total number of trials per class in each classification task, as well as the average, maximum and minimum number of instances available per participant (shown separately for appraisal check, class, and signal type).

**Table 1:** Number of instances available in the data sets obtained from Study 1 and Study 2 for the classification of single trials and averaged trials (Av.). Values are shown for both studies and signal types.

Study	Input signal	Number of instances	
		EEG	EMG
1	Single trials	16666	21529
	Av. of 2 trials	8222	10655
	Av. of 3 trials	5449	7074
	Av. of 4 trials	4071	5287
	Av. of 5 trials	3244	4232
	Av. of 10 trials	1583	2087
	Av. of 20 trials	753	1012
	Av. of all trials	150	138
2	Single trials	20185	18480
	Av. of 2 trials	9938	9100
	Av. of 3 trials	6590	6027
	Av. of 4 trials	4935	4527
	Av. of 5 trials	3921	3591
	Av. of 10 trials	1927	1770
	Av. of 20 trials	889	830
	Av. of all trials	192	176

**Table 2:** Number of instances available in each classification task. The values indicated are the total number of trials for each class in each classification experiment, as well as the average (Av.), maximum (Max.) and minimum (Min.) number of trials available per participant. Values are indicated separately for each signal type (EEG and EMG).

Signal	Study	Appraisal Check	Class	Total	Av.	Min.	Max.
EEG	1	Novelty	Familiar	12946	518	292	721
			Novel	3720	149	88	204
		Intrinsic Pleasantness	Unpleasant	5457	218	104	310
			Neutral	5574	223	141	307
			Pleasant	5635	225	135	311
	2	Control	High	15132	631	587	647
			Low	5053	211	191	216
		Goal Conduciveness	High	10087	420	392	431
			Low	10098	421	392	432
		Power	High	10098	421	392	432
			Low	10087	420	377	431
EMG	1	Novelty	Familiar	16766	729	633	721
			Novel	4763	207	182	202
		Intrinsic Pleasantness	Unpleasant	7206	313	274	310
			Neutral	7171	312	265	302
			Pleasant	7152	311	276	324
	2	Control	High	15138	631	577	647
			Low	5049	210	181	216
		Goal Conduciveness	High	10090	420	371	431
			Low	10097	421	387	432
		Power	High	10086	420	378	431
			Low	10101	421	380	432

We also explored the use of two subsets of EEG channels localized at specific scalp regions where effects of the appraisal checks were observed in the traditional EEG analyses of Studies 1 and 2. The specific EEG channels identified in the traditional analyses of the studies are shown in Table 3. The first (smallest) set corresponds to those EEG channels measuring the activity in the relevant localized scalp regions associated with the appraisal checks of each study. The second set includes the same channels plus all immediately neighbouring channels. Finally, the last set includes the full set of EEG channels. For each set of channels, we conducted classification experiments for all single trials and averaged trials input signals. It should be noted that Independent Component Analysis (ICA) could also have been used as a preliminary method for the identification of maximally temporally independent EEG signals in the full scalp data, which in turn could have been used to reduce the dimensionality of the EEG signal space. This is indeed a standard method used in traditional EEG analyses. Nonetheless, given the evidence from the original analysis results of Studies 1 and 2, we decided for a theoretically-driven selection (rather than a data-driven selection).

**Table 3:** Sets of EEG channels used in the classification experiments. For each study, the first (smallest) set comprises those EEG channels measuring activity in the specific regions where the effects of the appraisal checks were observed in the traditional EEG analyses of the studies. The second set includes the same channels plus all immediately neighbouring channels. Finally, the last set includes the full set of EEG channels.

	Study 1	Study 2
Set 1	Fz, Cz, Pz	Fz, FCz, Pz, POz
Set 2	Fz, Cz, Pz, FCz, F1, F2, AFz, C1, C2, CPz, P1, P2, POz	Fz, FCz, Pz, POz, F1, F2, AFz, FC1, FC2, Cz, P1, P2, CPz, PO3, PO4, Oz
Set 3	All 64 channels	All 64 channels

### Support vector machine classifiers

In our experiments, we applied Support Vector Machines (SVMs; e.g., [26]) for the participant-independent classification of the single trials and the averaged trials of the EEG (so-called ERPs) and EMG signals (frontalis, corrugator, and zygomaticus muscle regions) of the five appraisal checks investigated in Study 1 (novelty, intrinsic pleasantness) and Study 2 (goal conduciveness, control, and power).

SVMs are supervised learning models based on the concept of decision hyperplanes, that is, multi-dimensional boundaries that separate sets of objects with distinct class memberships. The goal of the SVM algorithm is to maximize the separation between classes, which consists of finding the hyperplane that has the largest distance to the nearest training data point of any class (also known as functional margin). Since the larger the margin, the lower the generalization error of the classification task. A set of training instances belonging to two or more categories are used to determine the hyperplane that best discriminates among different classes (i.e., that with the widest possible gap). The testing instances are then mapped onto this multi-dimensional space and the side of the gap they fall determines the predicted categories.

Formally, given a set of examples [*x*_*i*_, *y*_*i*_], *i* = 1, 2, …, *m*, where $x_{i}\in R^{d}$ is a *d*-dimensional feature vector, and *y*_*i*_ ∈ {0, 1} is a corresponding prediction of each example, the maximum margin separating hyperplane can be found by solving the following optimization problem:

$$\begin{array}{c} \begin{array}{cc} {\text{max}}_{\alpha }W\left(\alpha \right) & =\sum_{i=1}^{m}\alpha_{i}-\frac{1}{2}\sum_{i,j=1}^{m}y^{\left(i\right)}y^{\left(j\right)}\alpha_{i}\alpha_{j}K\left(x_{i},x_{j}\right)\\ \text{participant}\;\text{to:}\; & 0\leq \alpha_{i}\leq T,i=1,\ldots ,m\\ & \sum_{i=1}^{m}\alpha_{i}y^{\left(i\right)}=0, \end{array} \end{array}$$ (1)

where the $\alpha_{i}^{\prime }s$ that are Lagrangian multipliers satisfy the above constraints, *T* is a defined constant, and *K*(**x_i_**, **x_j_**) is a kernel function that can be linear, polynomial, radial basis, or sigmoidal. To classify a given test example, the following function is implemented:

$$\begin{array}{c} f\left(x\right)=\sum_{i}^{m}\alpha_{i}y_{i}K\left(x_{i},x\right)+b, \end{array}$$ (2)

where *b* is the ‘bias’ term that is often assumed to have zero mean. The sign of this function determines the category of the test example.

For the experiments reported in this paper, we used SVM with linear kernel functions as implemented in the WEKA toolkit [27], which uses the popular Sequential Minimal Optimization (SMO) [28] algorithm for solving the optimization problem during training. We chose SVM because this technique has matured theoretical foundations and has shown a remarkable performance on a variety of classification tasks over the years, including classification of physiological signals [29]. Furthermore, SVM have good generalization properties (e.g., [30, 31], are robust against overtraining [31] and to the curse-of-dimensionality [30, 32]. In particular, the last characteristic is especially relevant for the analysis of physiological signals since the dimensionality of the feature space is high and the training sets are relatively small. Other modelling techniques, especially those related to Deep Learning [33], also show a strong potential for this line of research, but a comparison and optimisation of ML techniques is beyond the scope of the present study and will be part of our future work.

### Feature extraction

We extracted a set of energy- and spectrum-related features from the EEG and EMG signals. Features were extracted on the complete signals (window size of 1,500 ms) obtained in each trial (or, depending on the classification experiment, the average signal across several trials) as well as segments of the signals determined by a sliding window with a size of 200 ms (EEG) and 400 ms (EMG) with 50% overlap. For the EEG signals, the sliding windows were only applied to the initial 1,000 ms (leading to a total of nine segments: 0-200 ms, 100-300 ms, 200-400 ms, 300-500 ms, 400-600 ms, 500-700 ms, 600-800 ms, 700-900 ms, and 800-1000 ms). For the EMG signals, they were applied over the whole signal (1,500 ms), which resulted in seven segments (0-400 ms, 200-600 ms, 400-800 ms, 600-1000 ms, 800-1200 ms, 1000-1400 ms and 1200-1500 ms; the last window is 100 ms shorter than previous given the signal length).

#### EEG features

For the entire signal segments (1,500 ms) and each of the nine 200-ms long sliding windows, we used rectangular windows with 10% Hanning fade to compute the log amplitudes of eight filter banks (i.e., arrays of band-pass filters that decompose each signal into multiple frequency components) tuned to logarithmic-scaled frequencies in the region between 1 and 40 Hz, as well as the root mean square (RMS) signal frame energy. A logarithmic scale was used in order to create a set of filter banks with more filters tuned to the lower frequencies (i.e., those frequencies where we expected to find the relevant signal information). Additionally, for the entire segments, we also computed the spectral centroid (i.e., the balancing point of the spectrum), the positions of minimum and maximum amplitudes, the signal entropy (i.e., the spectral complexity or irregularity), the standard deviation, and the slope (calculated from the same eight logarithmic-scaled filter banks). In total, 96 static features were extracted for each EEG channel.

#### EMG features

For the entire signal segments (1,500 ms) as well the seven 400-ms long sliding windows, we used again rectangular windows with 10% Hanning fade to compute the log amplitudes of another set of logarithmic scaled filter banks, and the RMS signal frame energy. In the case of the EMG signals, we used a set of 10 filter banks in the frequency range of 20-60 Hz. Similarly to the EEG signals, for the entire segments, we also computed the spectral centroid, the positions of minimum and maximum amplitudes, the entropy, the standard deviation, and the slope (calculated from the ten logarithmic-scaled filter banks). In total, 94 static features were extracted for each facial EMG region.

For both EEG and EMG signals, each instance is described by a static vector formed through concatenating all the extracted features for each channel used in each classification experiment (i.e., containing all features extracted for all channels used in each experiment). Given that the largest number of input signals in our experiments corresponds to the 64 (full scalp) EEG channels, the maximum size of the input vector in all classification experiments is 6144 (96 x 64). All features were extracted using openSMILE suite [34].

### Development and testing methods

To limit the over-fitting of the classifiers to the training data and participant-specific activity, we used a three-fold participant-independent nested cross-validation (SICV) schema. Each fold comprises all trials (or average across trials) obtained from one third of the participants. In each SICV fold, one partition is used for training the classifiers (training set), another for estimating the model parameters during the development phase (validation set), and the third partition for testing the classifier in the unsupervised phase with a new group of participants (test set). All sets were standardized to the mean and standard deviation of the training sets in each fold. Additionally, given that some of the class distributions are highly unbalanced (i.e., the number of instances belonging to each class is very different), upsampling of the minority classes was performed on the training set to achieve even class distributions. This was achieved by repeating the full set of instances of the minority class(es) so that the percentage of instances representative of each class in the training set is as similar as possible (ideally 50% for binary classifications and 33% for ternary). The complexity parameter C that regulates margin optimization (*C* ∈ 10 − 5, 10 − 4, 10 − 3, 10 − 2, 10 − 1) was optimized using the validation sets of each SICV fold. Then, for each fold, training and validation sets were concatenated and a new classifier was trained with the optimized parameter C estimated in the development phase. Finally, the classifiers developed for each SICV fold were tested on the respective test sets as well as nine bootstrapped sets sampled (with replacement) from the original test set (with the same number of instances). The whole procedure was repeated for all classification experiments (both signals types and trials averaged) reported in this paper.

### Performance measures

The classifiers’ performance was quantified using the unweighted average of the class-specific recalls (or Un-weighted Average Recall; *UAR*), which reflects the number of correctly classified instances. Since the theoretical chance level (100/*NumberofClasses*) assumes infinite sample sizes, the threshold of correct classification needs to be estimated in order to correctly interpret the difference between the *UAR* calculated and the actual chance level for each experiment. This is especially important in our study given the disparity between the number of test instances used in the classification experiments (see Table 1), and particularly the small number of instances in some of them (less than 50). In order to estimate the analytical chance levels, we used the method described in [35], that estimates the threshold that needs to be exceeded in order to consider the decoding statistically significant for different sample sizes using a binomial cumulative distribution. The difference between the *UAR* and the analytical chance level (*diffUAR*) for a specific test set size was then used to determine the actual performance of the classifiers relative to the analytical chance level at a 95% significance level.

## Results

The results for the classification experiments for each appraisal check by signal type (and number of channels used per signal; only for EEG) and number of trials averaged is shown in Tables 4 and 5 (EEG), and Tables 6 and 7 (EMG). The values shown correspond to the performance of the optimized classifiers on the test sets of the three SICV folds plus nine bootstrapped sets sampled (with replacement) from the original test sets (with the same number of instances; a total of 3 + 3 * 9 = 30 test sets per classifier). This method was used to obtain robust estimations of the algorithm on the test set data. Furthermore, by inferring the distribution of the test predictions, we can also apply inferential statistics to determine if they are significantly above the empirical chance level (using one-tailed Student’s *t*-tests for a single sample) and determine the configurations (channels and trials averaged) that lead to the best performances (using Linear Mixed Models (LMM)).

**Table 4:** Summary of the results pertaining the classification of the EEG signals in terms of *novelty* and *intrinsic pleasantness* appraisal checks manipulation (Study 1). Results are shown for different numbers of averaged trials (Av.) per participant, and different numbers of channels. The classifiers’ performance was quantified using the Unweighted Average Recall (*UAR*), and the difference between the UAR and the analytical chance level (*diffUAR*). Star symbols indicate significant one-tailed Student’s *t* -tests conducted to examine when classification performances were significantly above empirical chance level (***p < .001, **p < .01, *p < .05). For details on the number of trials averaged per participants see Table 2.

Number of Av. Trials	UAR			diffUAR		
	3 ch.	13 ch.	64 ch.	3 ch.	13 ch.	64 ch.
**Novelty**						
All^⊗^	80.2±8.9***	82.9±7.4***	82.3±6.2***	18.2±9.3	20.8±7.0	20.3±6.2
20	66.1±5.2***	71.6±2.7***	83.5±4.2***	10.8±5.1	16.4±2.8	28.3±4.4
10	63.6±3.8***	68.7±2.9***	76.8±3.8***	9.9 ±3.6	15.1±2.8	23.2±4.0
5	64.8±2.1***	63.6±2.0***	72.6±3.1***	12.3±2.0	11.0±2.1	20.1±3.2
4	61.4±1.6***	62.9±1.9***	71.4±3.1***	9.2 ±1.5	10.7±2.0	19.2±3.3
3	60.2±2.8***	62.3±2.7***	70.1±2.2***	8.3 ±2.7	10.4±2.7	18.2±2.2
2	59.2±1.2***	60.3±1.2***	67.9±2.3***	7.6 ±1.2	8.8 ±1.2	16.4±2.2
None	56.0±1.2***	58.6±1.3***	64.5±1.0***	4.9 ±1.1	7.5 ±1.3	13.4±1.0
**Intrinsic pleasantness**						
All^⊗^	33.5±6.2	36.1±8.8	37.9±7.1	-10.4±6.2	-7.9±9.0	-6.1±7.0
20	39.3±3.2*	37.1±3.0	37.2±2.3	1.0 ±3.4	-1.0±3.1	-1.1±2.3
10	36.2±1.9	33.1±1.9	36.3±1.9	-0.5 ±1.9	-3.7±1.8	-0.5±1.9
5	33.7±1.5	34.6±2.6	34.6±1.5	-2.0 ±1.5	-1.1±2.7	-1.1±1.5
4	34.2±2.0	36.5±2.3	36.4±1.6*	-1.2 ±2.1	1.1 ±2.4	1.0±1.7
3	33.8±1.7	34.6±1.9**	35.3±0.9	-1.4 ±1.8	-0.6±1.9	0.1±0.9
2	33.8±1.3	34.2±1.1	36.1±0.7*	-1.0 ±1.4	-0.6±1.1	1.3±0.7
None	34.1±0.7	34.5±0.9	34.8±0.5*	-0.2 ±0.7	0.2 ±0.9	0.4±0.5

**Table 5:** Summary of results pertaining the classification of EEG signals in terms of *control*, *power* and *goal conduciveness* appraisal checks manipulation (Study 2). Results are shown for different sizes of numbers of averaged trials per participant, and different numbers of channels (ch.). The classifiers’ performance was quantified using the Unweighted Average Recall (*UAR*), and the difference between the UAR and the analytical chance level (*diffUAR*). Star symbols indicate the significant one-tailed Student’s *t* -tests conducted to examine when classification performances were significantly above empirical chance level (*p < .05, **p < .01, ***p < .001). For details on the number of trials averaged per participants see Table 2.

Number of Av. Trials	UAR			diffUAR		
	4 ch.	16 ch.	64 ch.	4 ch.	16 ch.	64 ch.
**Control**						
All^⊗^	56.1±5.9	56.6±6.3	54.9±5.9	-4.8±5.9	-4.4±6.3	-6.0±5.9
20	50.2±4.7	55.0±3.3	48.7±4.3	-4.6±4.7	0.2±3.3	-6.1±4.3
10	50.8±2.7	51.4±3.3	51.4±2.1	-2.5±2.7	-1.9±3.2	-1.8±2.1
5	52.8±1.9	51.6±1.5	51.4±1.8	0.5±2.0	-0.7±1.5	-0.9±1.8
4	51.9±1.8	51.0±1.6	49.8±1.9	-0.2±1.8	-1.1±1.6	-2.2±1.9
3	51.0±1.4	51.5±1.6	48.7±1.3	-0.7±1.4	-0.2±1.6	-3.1±1.3
2	49.6±1.0	50.1±1.3	50.2±1.5	-1.8±1.0	-1.3±1.3	-1.2±1.5
None	50.4±0.6	49.7±0.6	49.5±0.7	-0.6±0.6	-1.3±0.6	-1.5±0.7
**Power**						
All^⊗^	70.6±5.3***	65.6±7.3*	59.1±6.0	9.7±5.3	4.7±7.3	-1.9±6.0
20	56.2±3.9*	55.2±3.1	54.1±3.2	1.5±4.0	0.5±3.1	-0.6±3.2
10	57.7±2.5***	56.2±2.9***	53.8±2.5	4.4±2.5	2.9±2.9	0.5±2.5
5	55.4±1.6***	55.7±1.5***	52.7±1.2	3.1±1.6	3.4±1.5	0.4±1.2
4	53.5±1.7***	54.0±1.6***	53.0±1.2 ***	1.4±1.7	1.9±1.6	0.9±1.2
3	54.2±1.2***	52.1±1.4	52.0±1.3	2.5±1.2	0.4±1.4	0.2±1.3
2	52.5±0.8***	51.8±1.1*	52.3±0.9 ***	1.0±0.8	0.4±1.1	0.9±0.9
None	51.9±0.5***	51.9±0.6***	50.7±0.9	0.9±0.5	0.9±0.6	-0.3±0.9
**Goal Conduciveness**						
All^⊗^	53.4±8.5	59.2±6.4	56.3±5.6	-7.6±8.5	-1.8±6.4	-4.6±5.6
20	56.6±3.6 **	55.4±3.0	55.4±3.1	1.8±3.6	0.6±3.1	0.6±3.1
10	54.9±2.0 ***	52.4±2.8	51.5±2.2	1.6±2.0	-0.9±2.8	-1.8±2.2
5	54.2±1.6 ***	53.1±1.7 **	52.1±1.1	1.9±1.6	0.8±1.7	-0.2±1.1
4	52.8±1.4 **	51.7±1.4	52.2±1.4	0.8±1.4	-0.4±1.4	0.2±1.4
3	53.3±1.1 ***	52.3±1.1 **	50.7±1.1	1.5±1.1	0.6±1.1	-1.0±1.1
2	51.8±0.9 *	51.9±0.9 **	52.2±1.0 ***	0.3±0.9	0.4±0.9	0.8±1.0
None	52.1±0.8 ***	51.9±0.6 ***	52.0±0.6 ***	1.1±0.8	0.9±0.6	1.0±0.6

**Table 6:** Summary of the classification results obtained for the *novelty* and *intrinsic pleasantness* appraisal checks from the EMG signals (Study 1). Results are shown for different numbers of averaged trials per participant. The classifiers’ performance was quantified using the Unweighted Average Recall (*UAR*), and the difference between the UAR and the analytical chance level (*diffUAR*). Star symbols indicate the significant one-tailed Student’s *t* -tests conducted to examine when classification performances were significantly above empirical chance level (*p < .05, **p < .01, ***p < .001).

Appraisal check	Number of Av. Trials	UAR	diffUAR
**Novelty**	All^⊗^	54.4±8.7	-7.9±8.7
	20	49.3±4.8	-5.2±4.7
	10	47.9±2.4	-5.2±2.4
	5	51.3±2.2	-0.9±2.2
	4	50.8±1.2	-1.2±1.2
	3	50.5±0.8	-1.2±0.8
	2	50.7±0.6	-0.7±0.6
	None	50.1±0.2	-0.9±0.2
**Intrinsic Pleasantness**	All^⊗^	31.2±5.8	-13.1±5.8
	20	33.4±2.7	-4.2 ±2.7
	10	32.0±1.5	-4.3 ±1.4
	5	33.9±1.6	-1.5 ±1.6
	4	33.7±1.0	-1.5 ±1.0
	3	32.8±0.7	-2.1 ±0.7
	2	31.7±1.1	-3.0 ±1.2
	None	33.6±0.6	-0.7 ±0.6

^⊗^ For details on the number of trials averaged per participants see Table 2.

**Table 7:** Summary of the classification results obtained for the control, goal conduciveness and power appraisal checks from the EMG signals (Study 2). Results are shown for different numbers of averaged trials per participant, and different numbers of channels (only for EEG). The classifiers’ performance was quantified using the Unweighted Average Recall (*UAR*), and the difference between the UAR and the analytical chance level (*diffUAR*) estimated using the method described in [35]. Star symbols indicate the significant one-tailed Student’s *t* -tests conducted to examine when classification performances were significantly above empirical chance level (*p < .05, **p < .01, ***p < .001).

Appraisal check	Number of Av. Trials	UAR	diffUAR
**Control**	All^⊗^	45.5±7.3	-15.2±7.3
	20	49.2±3.2	-5.7 ±3.2
	10	47.9±2.1	-5.4 ±2.2
	5	48.0±1.5	-4.4 ±1.6
	4	48.0±1.6	-4.1 ±1.5
	3	47.0±2.4	-4.9 ±2.4
	2	49.4±1.3	-2.1 ±1.2
	None	48.8±0.9	-2.3 ±0.9
**Power**	All^⊗^	53.1±6.1	-7.7±6.1
	20	52.0±3.4	-2.8±3.5
	10	50.5±2.4	-2.8±2.5
	5	50.4±1.2	-2.0±1.2
	4	50.5±1.2	-1.6±1.2
	3	49.8±1.2	-2.0±1.1
	2	49.1±1.1	-2.4±1.1
	None	48.5±0.7	-2.6±0.7
**Goal Conduciveness**	All^⊗^	49.9±7.1	-10.9±7.1
	20	49.8±3.7	-5.1±3.8
	10	49.4±1.9	-3.9±1.9
	5	50.4±2.0	-2.0±2.0
	4	52.5±1.3	0.4 ±1.3
	3	49.8±1.4	-2.1±1.4
	2	50.7±0.9	-0.8±0.9
	None	50.3±1.0	-0.8±1.0

^⊗^ For details on the number of trials averaged per participants see Table 2.

### EEG

The results from the classification experiments using EEG signals are shown in Tables 4 and 5.

#### Novelty

All classification tests were significantly (and largely) above empirical chance level (*p* < .001 in all cases). The results obtained indicate that averaging across a larger number of individual trials and using the information from all EEG channels is beneficial for the classification of the *novelty* appraisal check. The best performance (in terms of the difference relative to the empirical chance level) was achieved using features extracted from the whole scalp (64 channels), and by averaging across 20 individual trials in the same experimental condition (*diffUAR* = 28.3 ± 4.4; *UAR* = 83.5 ± 4.2). The worst performance was achieved in the classification of single trials and using the features extracted from the three channels of interest (*diffUAR* = 4.9 ± 1.1; *UAR* = 56.0 ± 1.2), although even here performance was significantly (but only modestly) above chance level.

#### Intrinsic pleasantness

Only a few classification tests yielded results significantly above empirical chance level, and those that did were very modest (around 1% above empirical chance level).

#### Control

None of the tests resulted in classification performances above the respective empirical chance level.

#### Power

All classification tests using the features extracted from the four EEG channels of interests for this task yielded performances significantly above the empirical chance level. There is an apparent trend indicating that the performance improves when the signals being classified are those averaged across a larger number of trials (i.e., those with a higher SNR). The results are similar to the tests that used features extracted from 16 channels, although for the tests using signals averaged across three and 20 individual trials the performances are not significantly above the empirical chance level. The results obtained using all EEG channels are either not significantly above chance level, or only marginally. Overall, the best performance was obtained by using the signals from the four EEG channels of interest averaged across all trials obtained for each individual in the *Power* condition (*diffUAR* = 9.7 ± 5.3).

#### Goal conduciveness

All classification tests using features extracted from the four channels of interest yielded results significantly above empirical chance level (with the exception of all trials averaged), but only by a small margin (maximum of 1.9%). Only a few tests that used features from 16 or 64 EEG channels yielded results above empirical chance level, and by no more than 1%. The best results for this appraisal check was obtained using features extracted from the four EEG channels of interest, and the signals averaged across five individual trials.

### EMG

None of the tests resulted in classification performances above the respective empirical chance level for any of the appraisal checks studied in this paper.

## Conclusions

In this article, we have applied Machine Learning methods with the aim of finding evidence of psychobiological markers of emotion processes in EEG and EMG signals. In particular, we focused on determining whether various stages of event evaluation (appraisal) as postulated by appraisal theories can be automatically detected in this type of psychophysiological signals. Additionally, we attempted to determine the ideal number of trials of an experimental condition necessary for a successful classification of appraisal checks, as well as the usefulness of signals from localized activity over specific scalp regions of interest rather than the whole scalp.

Our results have shown that brain activity (EEG signals) allows clearly to detect the signal related with *novelty* and *power* appraisal checks. Indeed, we were able to achieve a classification accuracy of up to 85.5% and 70.6% (respectively) in this (binary) task. These results are even more striking if we consider that we have developed participant-independent models, that is, models developed with data from a subgroup of participants and generalized to a new group of participants. This indicates that our method permits detecting *novelty* and *power* appraisal checks in EEG signals, and that the model can be applied to new participants with similar characteristics to the sample used in this work (i.e., young, right-handed students who are in good health) without the need to adapt it. To a lesser extent, we also found evidence in brain activity for the *goal conduciveness* appraisal check. The best classification accuracy obtained for this check was 56.6%, however, this value is only 1.8% above the empirical chance level (54.8%). Our method did not allow us to detect *intrinsic pleasantness* and *control* appraisal checks sufficiently well. As these latter checks have been found using traditional EEG analyses, in the studies for which these data sets were originally collected [17, 18], this suggests that different feature sets and/or ML methods may be necessary for their automatic detection.

It should be noted that data interpolation from bad channels could potentially affect the objective to test the number of required data channels, as interpolated channels are defined by surrounding neighbours. However, our finding that classification results were generalisable across participants (i.e., results were consistent while the number and location of the interpolated channels varied across participants) suggests otherwise. Furthermore, we checked the total number of interpolated channels per data set and per set of EEG channels and we observed that a very small number of channels were interpolated in both studies (see data sets section). This suggests that interpolation was mild and not containing systematic biases.

In relation to the EMG data, we were not able to detect signals related with the appraisal checks under study. In all cases, the classification accuracy fell below the respective empirical chance levels. Given that appraisal effects have been found in the EMG data of Study 2 using traditional analysis methods, one possible explanation for this null finding may be that different feature sets may be necessary for the automatic detection of appraisals in EMG. Indeed, given the lack of information regarding the temporal location of the effects in the post-stimulus phase, we have focused on extracting features from the whole signals as well as seven large temporal windows, but it may be that these time intervals are not adequate. Several studies show that the effect of appraisals in EMG are not stable over time, and may result in significant differences between conditions only in brief (e.g., 100 ms) time windows during the post-stimulus interval (see e.g. [36–38]). Although we tried to capture this non-stationarity in the present ML-based study by segmenting the EMG data into shorter time windows, we chose 400-ms time windows in order to limit the overall number of tests. These windows may have been too large to detect subtle (i.e., more temporally fine-grained) appraisal effects. Future work should address this issue. It should also be noted that previous work has not investigated the effects of all of the currently investigated appraisal checks in EMG activity. It is therefore possible that the effects of some appraisal checks (e.g., Study 1) are not mirrored in facial muscle activity changes (or at least hard to detect). Furthermore, the results may be affected by the interaction between subsequent checks. Indeed, the EMG data analysis performed by Gentsch and colleagues [36](Experiment 1) indicates that integrated information related to goal conduciveness and power triggers cheek muscle activity changes. Similarly, van Peer and colleagues [17] found that some intrinsic pleasantness effects in the EEG data were affected by novelty. It is possible that these appraisal check effects could not be detected in the current work due to the fact that we have classified all appraisal checks in isolation (in order to be able to use all available trials for each appraisal check).

It is important to highlight that the number of available data samples to train the models (i.e., the training instances) was vastly altered when different numbers of trials were averaged. This is potentially problematic given that Machine Learning is highly sensitive to the sample size of the training data set. In principle, the models can learn more from a larger training set size, which can lead to superior performance. To mitigate this problem, we have explicitly considered the size of the data set as a factor in the statistical analyses, through the calculation of empirical chance levels based on the number of instances in each classification task (more trials averaged meant less instances). The results for each classification task were based on the difference between its performance and the empirical chance levels, which allowed to compare the performance of the various tasks in an unbiased way. Our findings thus show that classification performance is robust across different numbers of trials. Furthermore, our results show that the best performance was achieved by the models with smaller training sets (more trials averaged). This is most likely due to the higher signal-to-noise ratio (SNR) of these averaged signals, and suggests that the SNR may affect the results more than the training set size.

The proposed ML methodology is a promising tool for the development of computerized tools (e.g., Brain-Computer Interface) that, combined with appropriate tools for automatic pre-processing of the raw signals, can be applied to the study of cognitive processes central for the elicitation and differentiation of emotional episodes. In particular, it provides a potential avenue to explore the brain and efferent physiological correlates of specific emotion-related cognitive processes, and their application to the study of the mechanisms underlying (general or pathological) emotional responses. In this context, we have shown that Machine Learning offers viable tools to discriminate appraisal checks from central physiological signals without requiring *a priori* decisions about the analyses of the EEG recordings (e.g., choice of models or channels of interest). This is a great advantage compared to more traditional EEG analysis methods, as for many emotion processes the specific psychophysiological markers are not known yet. Additionally, we have shown that a robust discrimination can also be achieved using EEG signals averaged over only a small number of trials, which shows the potential for reducing the efforts associated with long recording sessions (which are often not readily feasible with babies, children, or clinical populations) and minimizing possible effects of habituation and learning. However, it should be noted that, in our classification experiments with single trials and a small number of averaged trials, the signals were collected in the context of a long recording session, and therefore effects of fatigue, habituation, or learning may be present in our data and may confound findings. It is possible that these effects masked the appraisal effects under investigation, which in turn may affect the features extracted and limit the classifiers to detect relevant properties of the signals that would permit a successful classification. Future studies are necessary to confirm that our results from the small trial sets can indeed be generalized to experiments with a short recording session, in which fatigue and habituation are assumedly negligible. Furthermore, is it important to note that the conclusions about the optimal number of trials and electrodes may be specific to the behavioural tasks that were applied, and cannot be readily generalized to other types of experimental paradigms, as the optimal values may differ, for example, due to the magnitude of the EEG signal relative to the background noise, or the spatial characteristics of the EEG patterns. Also, the data sets used in this study include a relatively small number of participants—a larger number would be necessary to unequivocally demonstrate the scalability of the proposed methods to a larger number of individuals.

Ongoing work focuses on the identification of temporal physiological patterns in EEG and EMG signals associated with the sequential nature of appraisal checks as predicted by the CPM, which could in the future potentially reveal more information about the type and intensity of the elicited emotion(s).
